# Pulmonary Artery Sarcoma With Lung Metastasis

**DOI:** 10.1002/rcr2.70534

**Published:** 2026-03-02

**Authors:** Ryoju Sato, Tomoki Teramoto, Masamitsu Hamakawa, Toshihide Yokoyama, Tadashi Ishida

**Affiliations:** ^1^ Kurashiki Central Hospital, Respiratory Medicine Kurashiki Japan

**Keywords:** palpitation, pulmonary artery, pulmonary hypertension, sarcoma, tachycardia

## Abstract

Pulmonary artery sarcoma is a rare disease that has been frequently misdiagnosed. A 77‐year‐old woman presented with palpitations and was found to have pulmonary hypertension. Contrast‐enhanced computed tomography showed a characteristic contrast defect occupying the entire lumen of the main pulmonary artery, leading to a diagnosis of pulmonary artery sarcoma.

## Graphical abstract

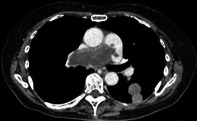

A 77‐year‐old woman presented with a one‐month history of palpitations. The electrocardiogram showed sinus tachycardia with a heart rate of 109 beats/min. Echocardiography demonstrated pulmonary hypertension with a tricuspid regurgitation pressure gradient (TRPG) of 67 mmHg. A chest radiograph showed a mass shadow in the left lung field (Figure 1A). Contrast‐enhanced computed tomography (CT) showed a contrast defect occupying the entire lumen of the main pulmonary artery and a mass shadow in the left lower lobe (Figure 1B). Contrast‐enhanced magnetic resonance imaging (MRI) showed a mass lesion with contrast enhancement in the main pulmonary artery (Figure 1C). Positron emission tomography (PET) exhibited increased uptake in the main pulmonary artery (Figure 1D). A CT‐guided biopsy of the left lung mass confirmed metastasis from pulmonary artery sarcoma (Figure 2).

**FIGURE 1:**
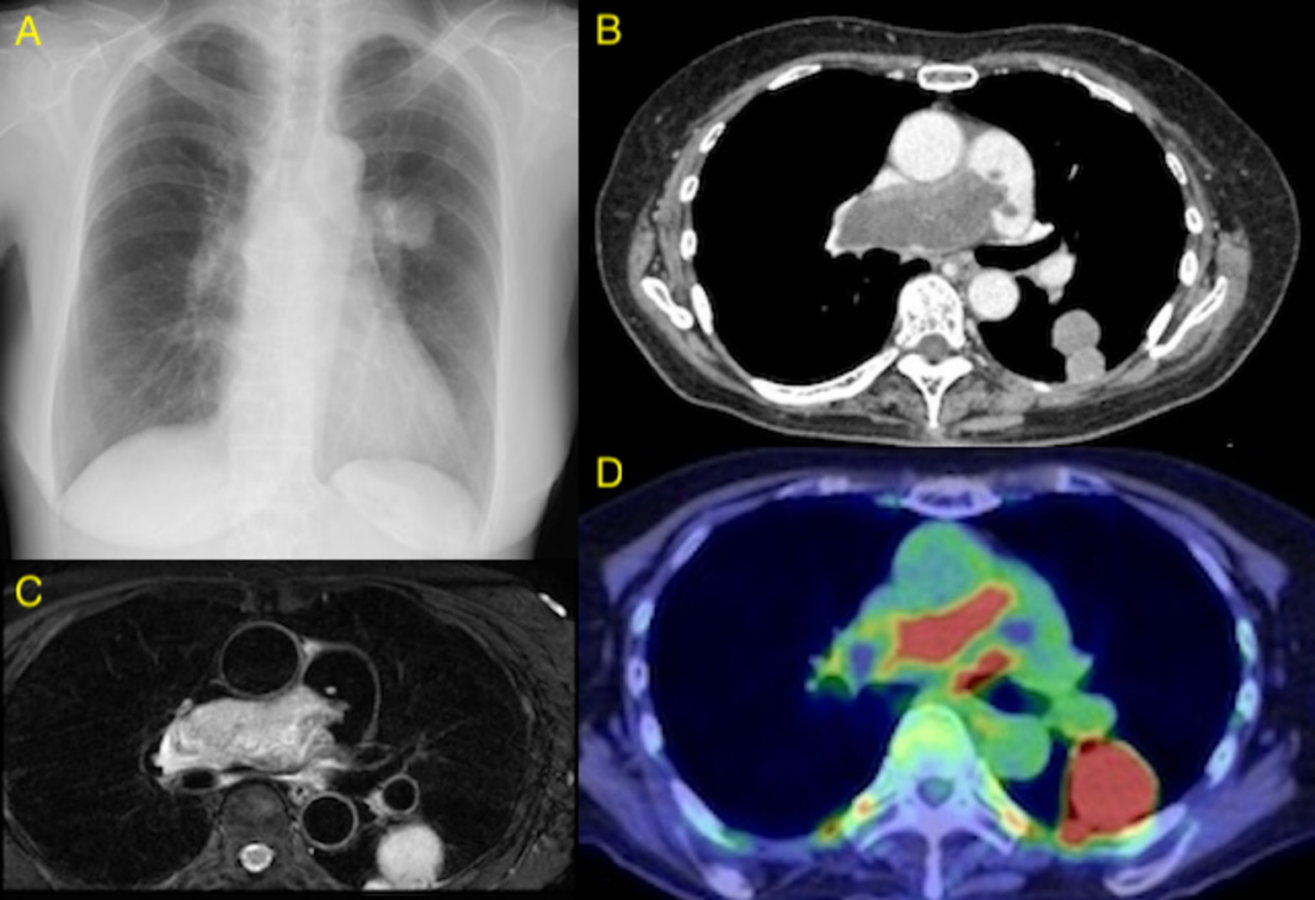
(A) Chest radiograph showing a mass shadow in the left middle lung field. (B) Contrast‐enhanced computed tomography showing a contrast defect occupying the entire lumen of the main pulmonary artery and a mass shadow in the left lower lobe. (C) Contrast‐enhanced magnetic resonance imaging showing a mass lesion in the main pulmonary artery, characterised by high signal intensity on T2‐weighted images with internal contrast enhancement. (D) Positron emission tomography showing standardised uptake value (SUV) max = 13.6 in the main pulmonary artery with areas of poor uptake within the lesion.

**FIGURE 2:**
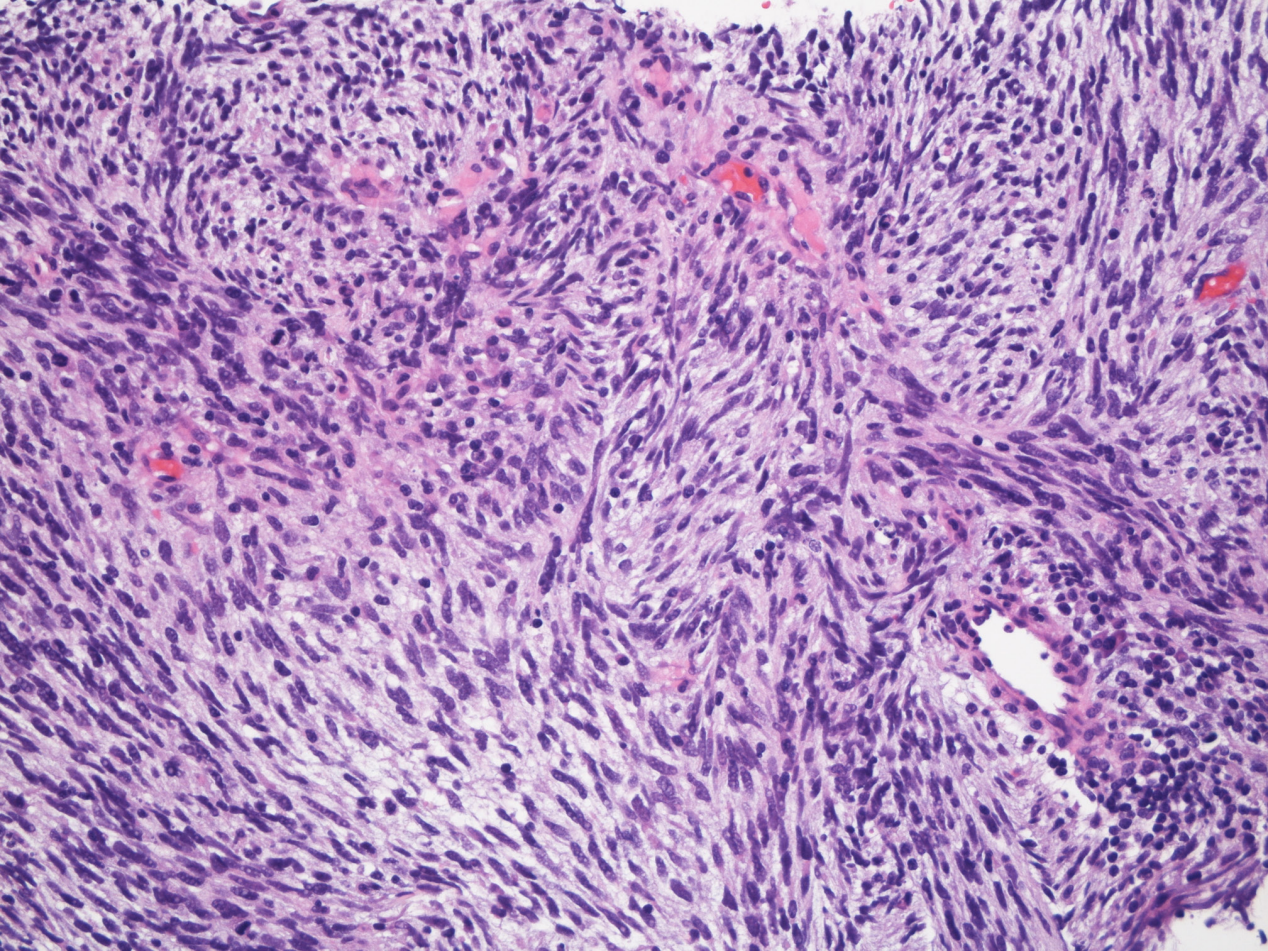
Malignant spindle cells with dense nuclei and basophilic cytoplasm proliferate in a fascicular pattern on Haematoxylin & Eosin images.

Pulmonary artery sarcoma was frequently misdiagnosed as pulmonary vascular diseases such as chronic thromboembolic pulmonary hypertension (CTEPH) or acute pulmonary embolism. Diagnosis requires chest contrast‐enhanced CT and cardiac ultrasound. Pulmonary artery sarcoma shows higher contrast enhancement than thrombi on contrast‐enhanced MRI and exhibits higher uptake than thrombi on PET. Clinicians should be familiar with the characteristic imaging found on contrast‐enhanced CT: a heterogeneous, low‐density filling the pulmonary artery with irregular distribution [1].

## Author Contributions

Ryoju Sato served as the attending physician for this patient and wrote this manuscript. Tomoki Teramoto also treated this patient. Masamitsu Hamakawa, Toshihide Yokoyama, and Tadashi Ishida supervised the patient's care and revised this manuscript.

## Consent

The authors declare that written, informed consent was obtained for the publication of this manuscript and accompanying images and attest that the form used to obtain consent from the patient complies with the Journal requirements as outlined in the author guidelines.

## Conflicts of Interest

The authors declare no conflicts of interest.

## Data Availability

The data that support the findings of this study are available on request from the corresponding author. The data are not publicly available due to privacy or ethical restrictions.
